# The p53 Tumor Suppressor-Like Protein nvp63 Mediates Selective Germ Cell Death in the Sea Anemone *Nematostella vectensis*

**DOI:** 10.1371/journal.pone.0000782

**Published:** 2007-09-12

**Authors:** Sandra Pankow, Casimir Bamberger

**Affiliations:** 1 Department of Cell Biology, The Scripps Research Institute, La Jolla, California, United States of America; 2 Sundgauallee 64, Freiburg, Germany; Fred Hutchinson Cancer Research Center, United States of America

## Abstract

Here we report the identification and molecular function of the p53 tumor suppressor-like protein nvp63 in a non-bilaterian animal, the starlet sea anemone *Nematostella vectensis*. So far, p53-like proteins had been found in bilaterians only. The evolutionary origin of p53-like proteins is highly disputed and primordial p53-like proteins are variably thought to protect somatic cells from genotoxic stress. Here we show that ultraviolet (UV) irradiation at low levels selectively induces programmed cell death in early gametes but not somatic cells of adult *N. vectensis* polyps. We demonstrate with RNA interference that nvp63 mediates this cell death *in vivo*. Nvp63 is the most archaic member of three p53-like proteins found in *N. vectensis* and in congruence with all known p53-like proteins, nvp63 binds to the vertebrate p53 DNA recognition sequence and activates target gene transcription *in vitro*. A transactivation inhibitory domain at its C-terminus with high homology to the vertebrate p63 may regulate nvp63 on a molecular level. The genotoxic stress induced and nvp63 mediated apoptosis in *N. vectensis* gametes reveals an evolutionary ancient germ cell protective pathway which relies on p63-like proteins and is conserved from cnidarians to vertebrates.

## Introduction and Results

All known organisms respond to genotoxic stress and have adopted strategies to cope with DNA damage. A distinctive feature of the stress response in multicellular eukaryotes is programmed cell death, or apoptosis, which eliminates damaged cells. Although apoptosis and its signal transduction mechanisms are very well described in vertebrate somatic cells [1], little is known about its regulation in germ cells. In particular, adverse environmental conditions like enhanced oxidative stress, UV irradiation, or nutrition deprivation–all conditions known to directly induce DNA damage or hamper its repair–result in high germ cell loss. This “death by defect” is commonly observed in the germ line in a variety of species across the animal kingdom and might be a selective mechanism for viable gametes [2]. However it is unclear how this selection is governed and whether, for example, p53-like proteins play a role in controlling this response. The tumor suppressor protein p53 is a key molecule in regulating the cellular response to genotoxic stress in somatic cells [1] and is mutated in more than 50% of all human tumors [3]. As “guardian of the genome”, p53 prevents the acquisition of new mutations during DNA repair and thus protects the integrity of the genome [4]. The evolutionary origin of its pivotal function has remained enigmatic and the discovery of two p53 siblings in vertebrates–p63 and p73–further added complexity to this question because of their high functional diversity. P73 is involved in a variety of processes ranging from nervous system development to governing inflammation [5], [6] whereas p63 regulates the proliferative potential of the epidermis [7]–[9]. Only very recently, first hints were provided that mammalian p63 also plays a pivotal role in controlling genome integrity as it specifically protects the female germ line from DNA damage [10]. Moreover, the question was raised of whether the genome protective function of p53 in somatic cells originates from an ancestral germ cell selecting mechanism that is controlled by p63-like proteins [10].

Apoptotic regulatory mechanisms have been extensively described in vertebrates and select invertebrate model organisms [11]–[14]; however, investigations into apoptosis in non-bilaterians has started only recently. Initial investigations revealed the existence of programmed cell death in the fresh water cnidarian *Hydra* [15]–[20] but the main regulatory mechanisms remained elusive [21]. To further decipher apoptotic regulatory mechanisms in non-bilaterians, we chose to study the starlet sea anemone *Nematostella vectensis*, which is a model organism belonging to the class of Anthozoa within the phylum Cnidaria [22]. Cnidaria are regarded as a sister group to the bilaterian metazoans and are commonly thought to be closely related to the ur-eumetazoa, which gave rise to Bilateria and Cnidaria. *N.vectensis* is extensively investigated as model organism for embryonic development [23], [24] and recent sequencing of its genome revealed a surprisingly high similarity to the human genome [25]. Thus results obtained from this model organism may be particularly informative with regard to the early evolution of apoptotic regulatory processes in bilaterians.

**Figure 1:**
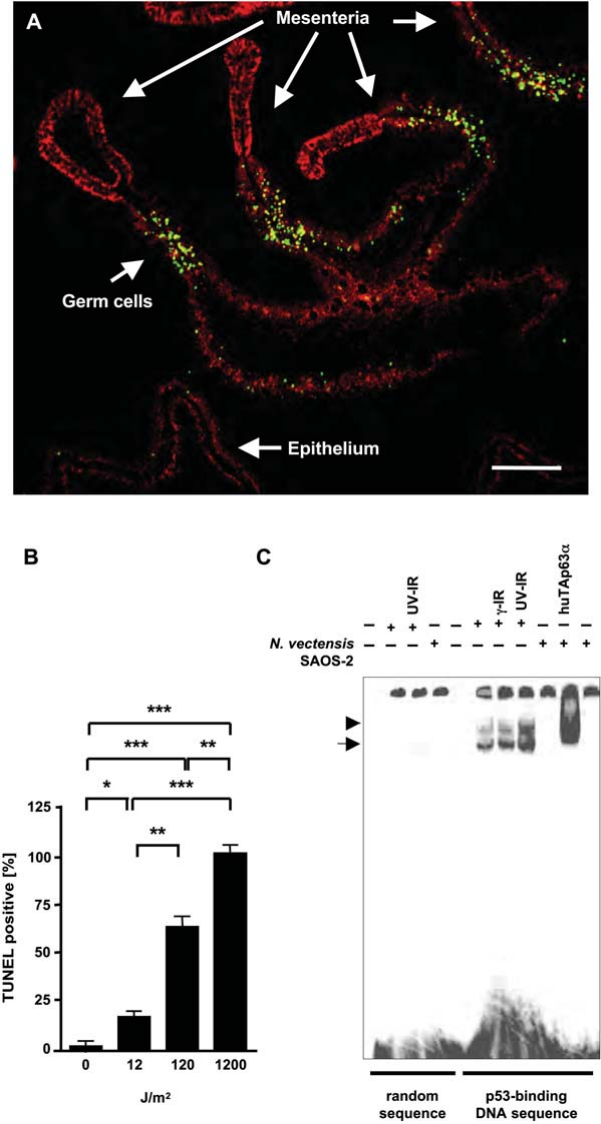
UV-induced germ cell death in adult starlet sea anemones. A The immunofluorescence image depicts the germ cell compartment of the mesenteria in a transverse section of an adult polyp. The epithelium of the body column consisting of ectoderm and endoderm is visible in the lower left corner. TUNEL staining (green) indicates the number of apoptotic cells in the mesenteria of adult polyps after 1200 J/m^2^ UV irradiation. Massive fragmentation of genomic DNA is detected in the germ cell compartment, whereas only few TUNEL positive cells were visible in the epithelium. Genomic DNA is stained with propidium iodide (red). Scale bar 150 µm. B Cell death in the germ cell compartment was quantified following different doses of UV irradiation by counting all TUNEL-positive gametes versus the total number of gametes. Error bars: standard deviation; * equals P<0.05, ** equals P<0.01, *** equals P<0.001. C Electromobility shift assay (EMSA) with whole protein lysates of irradiated (UV- or γ-IR) or non-irradiated adult *N. vectensis* polyps revealed DNA-binding activity for the canonical p53 binding sequence. Lysate was either incubated with a radioactively labeled control oligonucleotide with random DNA sequence (left section of the EMSA) or with a radioactively labeled oligonucleotide containing the consensus p53 DNA binding sequence (right section of the EMSA). Two different protein complexes (arrow and arrowhead) were detected using whole animal lysates from adult polyps when incubated with the p53 DNA binding sequence encoding oligonucleotide. SAOS-2 cells transfected with human TAp63α or vector only were analyzed in parallel as controls.

*N. vectensis* is exposed to varying levels of solar UV irradiation in its native habitat, the estuarine salt marshes along the Atlantic and North Pacific coasts. In order to investigate the response of the starlet sea anemone to genotoxic stress, we irradiated sexually mature adult polyps with increasing doses of UV light and determined the number of apoptotic cells. DNA fragmentation, a hallmark of programmed cell death, was detected with terminal deoxynucleotidyl transferase (TdT)-mediated dUTP nick end labeling (TUNEL). We found that high UV doses (1200 J/m^2^) induced massive apoptosis in the germ cell compartment (Fig. 1A). The germ cell compartment resides within the mesenteria [26], which together with the epithelium constitute the body column of adult *N. vectensis* polyps. TUNEL-positive cells were larger in size than the surrounding cells and displayed nuclei of high genomic DNA content. The same cells were also observed in non-irradiated adult polyps and have been previously described as early gametes in Anthozoa [27]. The number of apoptotic gametes was dependent on the dose of UV irradiation delivered: 12 J/m^2^ induced cell death in about 20% of all early gametes, 120 J/m^2^ eliminated more than 60%, and at a dose of 1200 J/m^2^ all early gametes were apoptotic (Fig. 1B). Almost none of the somatic cells present in the same tissue compartment or in the adjacent epithelium consisting of both ectoderm and endoderm responded with cell death at these doses.

In vertebrates the transcription factor p53 is a key protein that activates apoptosis in response to excessive DNA damage. Since p53-like proteins have been identified so far only in bilaterians [28], the evolutionary origin of this function has remained enigmatic. In light of the hypothesis that an ancient germ cell selective mechanism is mediated by transcription factors that are p63-like proteins, we asked how non-bilaterians control apoptosis and whether or not p53-like proteins would also be present in *N. vectensis*. To test for p53-like transcription factors in adult polyps *in vivo*, we performed electrophoretic mobility shift assays (EMSA) with whole animal lysates and radioactively labeled oligonucleotides containing either a random DNA sequence (Fig. 1C, left part of the EMSA) or the consensus p53 responsive element first described by El Deiry *et al.* [29] (Fig. 1C, right part of the EMSA). With this assay we detected two different protein complexes (Fig. 1C, arrow and arrowhead) that bound the consensus p53 responsive element (RE) but not the random DNA oligonucleotide. Human TAp63α transfected in human SAOS-2 cells was analyzed in parallel to verify the functionality of the assay. Furthermore, we investigated the consequence of either γ- or UV irradiation on the DNA binding of these two protein complexes. UV irradiation but not γ-irradiation of adult polyps enhanced DNA binding of the slower migrating protein complex (Fig. 1C, arrowhead) as shown by increased signal intensity. In contrast, the faster migrating complex (Fig. 1C, arrow) remained unaltered. The EMSA thus indicated that proteins or protein complexes that bound the p53 RE are present in adult polyps and are selectively induced after UV irradiation.

**Figure 2:**
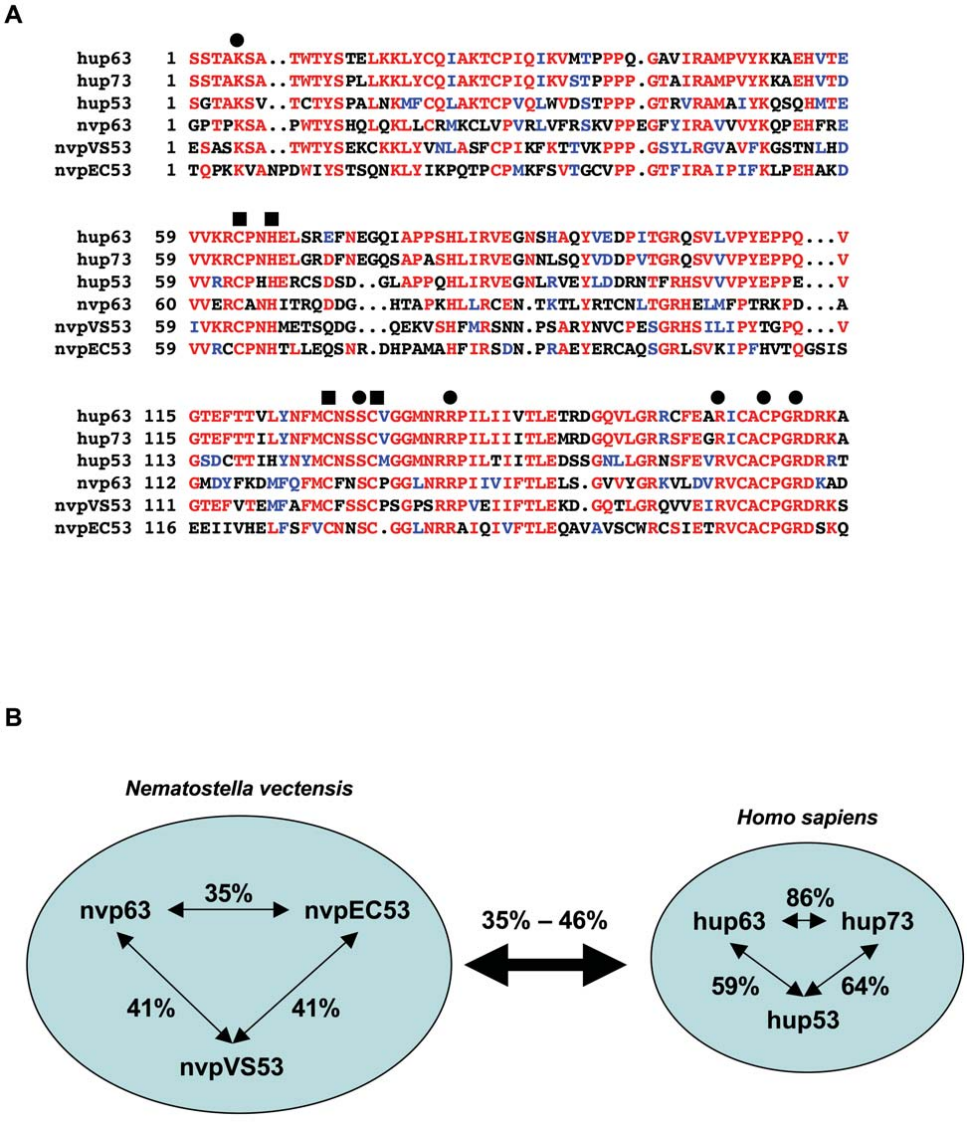
Protein sequence comparisons of the DNA binding domains of the human and *N. vectensis* p53 protein family. A Protein sequence comparison of the DNA binding domain sequences between the human (hu) and *N. vectensis* p53 protein family (nv). Black circles indicate all amino acids with direct contact to DNA identified in human p53 and black rectangles mark amino acids that complex a zinc atom. All of these amino acids are conserved in all *N. vectensis* p53 paralogs. B Protein sequence identities of the DNA binding domain within the p53 protein family of the species *H. sapiens* and *N. vectensis* are depicted. The thick double arrow indicates the range of protein sequence identities of all possible cross species sequence comparisons.

**Figure 3:**
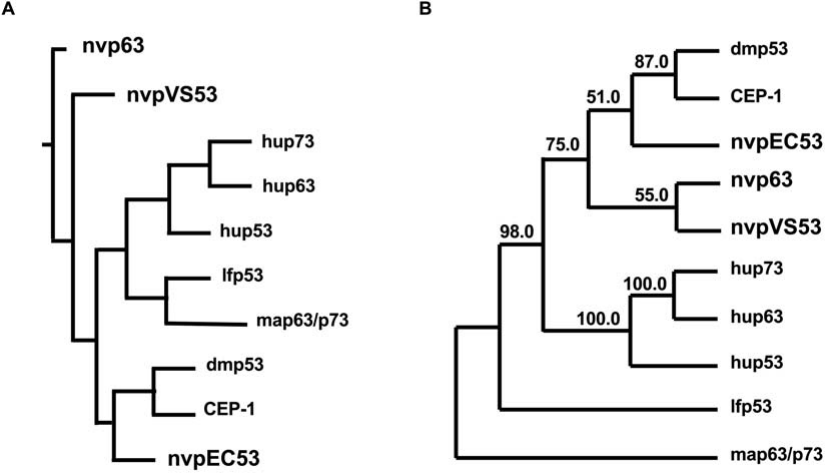
Phylogeny of p53-like proteins. A Schematic depicting a rooted phylogenetic tree for the p53 protein family of different species (nvpEC53, nvp63, nvpVS53: *Nematostella vectensis*, Cnidaria. Hup53, hup63, hup73: *Homo sapiens*, Deuterostomia. Lfp53: *Loligo forbesi*; map63/p73: *Mya arenaria*, Ecdysozoa. Dmp53: *Drosophila melanogaster* and CEP-1: *Caenorhabditis elegans*, Lophotrochozoa). B An unrooted phylogenetic tree with bootstrap values from 1000 iterations is depicted.

In contrast to the single p53-like homolog found in classical bilaterian invertebrate model organisms like *D. melanogaster and C. elegans*, RT-PCR and bioinformatic approaches identified three homologs of the vertebrate tumor suppressor protein p53 in this cnidarian. The three homologs were named nvp63, nvpEC53 for ecdysozoan-like p53 homologue, and nvpVS53 for very short p53 homolog (Fig. 2, 3). Full length cDNAs for all three family members were obtained by 5′ and 3′ RACE experiments with total RNA preparations isolated from adult polyps (nvp63 GI: DQ632751; nvpEC53 GI: EF424412; nvpVS53 GI: EF424410-1).

The core DNA binding domain is invariably present in all known p53-like transcription factors and its sequence similarity ranges from 37% to 42% within the starlet sea anemone p53-like protein family (Fig. 2A, B). This is lower than the percentage of amino acid identities observed within the human p53 protein family (59% to 86%). Whereas the vertebrate p53, p63 and p73 evolved from one ancestral p53-like protein during evolution of Deuterostomia, the three *N. vectensis* p53-like proteins evolved independently in cnidarians. Cross-species comparison of the two protein families revealed that *N. vectensis* p53-like paralogs are 35% to 46% identical to the human p53 protein family (Fig. 2B). In contrast, CEP-1 in *C. elegans* [12] and dmp53 in *D. melanogaster* [11] are only 18% and 27% identical to the human p53 protein family.

Phylogenetic sequence analysis using a consensus model tree with additional p53-like proteins from other species and covering the full length protein sequences support the suggested evolution of the vertebrate p53 protein family (Fig. 3A). All three *N. vectensis* paralogs root at the base of the phylogenetic tree. The bootstrap values in the un-rooted phylogenetic tree reflect the divergence of the *N. vectensis* protein family (Fig. 3B). Whereas nvpEC53 is allocated to the single p53-like proteins found in the ecdysozoans *C. elegans* or *D. melanogaster*, the third p53-like protein family member in *N. vectensis*–nvpVS53–branches off separately in the consensus tree.

**Figure 4:**
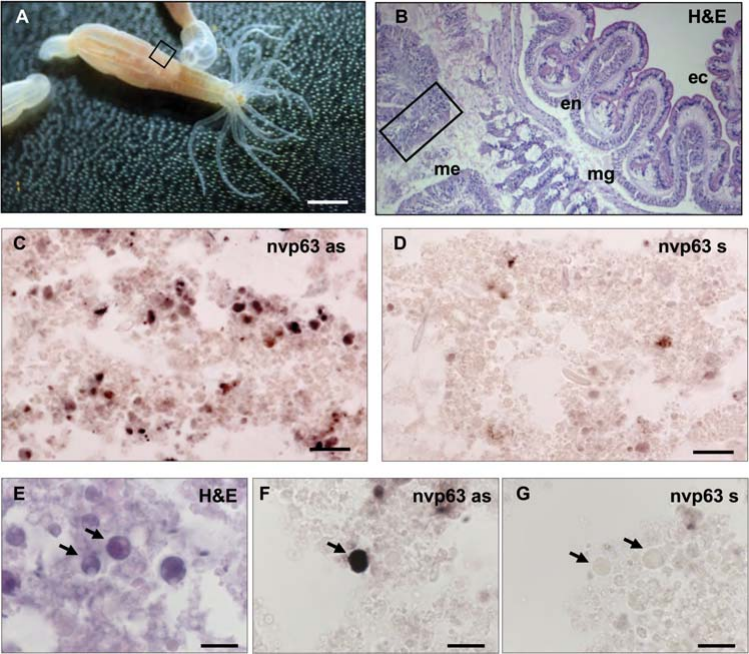
Nvp63 localizes to gametes. A–G Non-radioactive *in situ* hybridization revealed nvp63 expression in mesenterial gametes. A Adult *N. vectensis* polyps were sectioned longitudinally in the middle of the body column. B The Hematoxylin and Eosin staining (H&E) of the boxed region in A depicts the three prominent tissues in anthozoa: Ectoderm (ec) and entoderm (en) are separated by a mesoglea (mg). The mesenteria (me) reside within the body cavitiy of the polyps. C Strong hybridization of the antisense riboprobe was observed within mesenteria (region according to boxed area in B). D The hybridization signal was absent in serial sections incubated with the corresponding sense probe. Higher magnification of nvp63-positive cells indicated that a sub-population of larger, nearly round cells (E) express high levels of nvp63 (F), which is absent in control hybridizations (G). Arrows indicate round shaped gametes. Scale bars: A 0.5 cm; C,D 50 µm; E–G 10 µm.

*In situ* hybridization indicated that nvp63 is almost exclusively present in the mesenteria of adult *N. vectensis* polyps (Fig. 4A–G). Strongest expression of nvp63 mRNA was detected within the germ cell compartment of adult polyps. The same subset of round shaped early gametes, which undergo cell death upon UV irradiation, were also positive for nvp63 mRNA expression (Fig. 4C,F). These early gametes are distinct from small stem cells expressing the dead-box helicase protein vasa (nvvasa) in the germ line of *N. vectensis* [30]. Based on the highly specific localization of nvp63 mRNA, we further analyzed the nvp63 gene, cDNA and protein.

**Figure 5:**
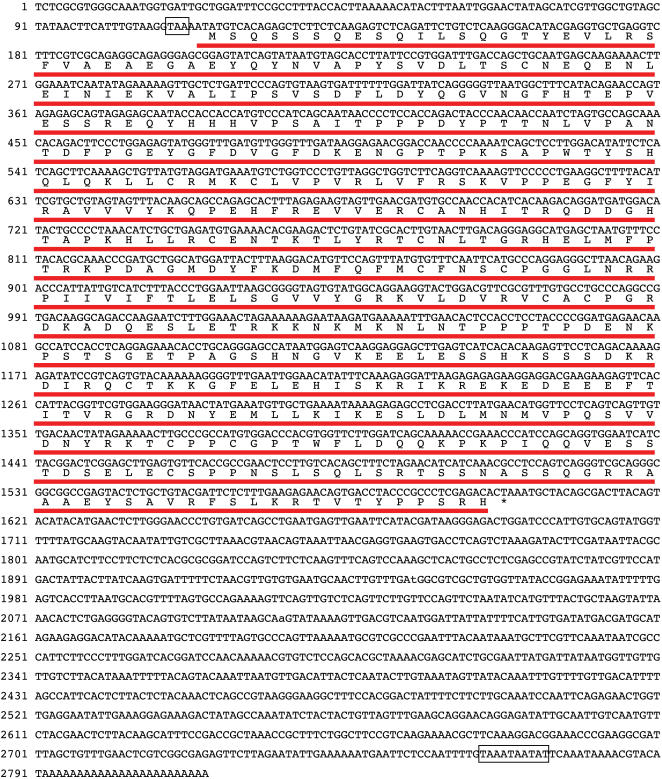
Nvp63 cDNA sequence. The mRNA for nvp63 comprises 158 bp of 5′-untranslated region with an in-frame stop codon preceding the start-methionine, a coding DNA sequence of 1485 bp (495 amino acids) length, and 1200 bp of 3′ UTR terminated by a polyadenylation signal. The open reading frame encoding the nvp63 protein is underlined in red. The stop codon preceding the open reading frame in the 5′ UTR and the putative polyadenylation signal at the end of the 3′ UTR are boxed.

**Figure 6:**
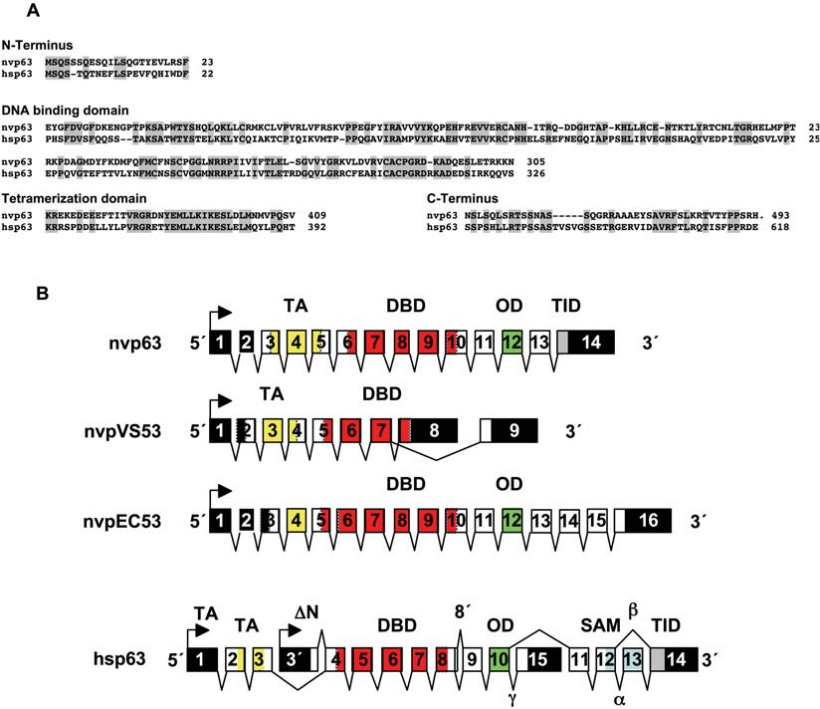
Protein comparison of nvp63 to hup63 and gene structure comparison of *N. vectensis* p53 protein family to hup63. A Protein sequence comparison between the human TAp63α (hup63) and nvp63. Highly identical regions comprise the N-terminus, the core DNA binding domain, the tetramerization domain, and the C-terminus. Several SQ motifs are present within the first 23 amino acids at the N-terminus. These motifs were identified in vertebrates as possible phosphorylation sites for DNA damage-induced kinases ATM or ATR [50], [51]. This potentially regulatory part of the protein is followed by a glutamate/aspartate rich amino acid sequence which is common to transcriptional activation domains (not shown in sequence comparison). Like in other invertebrates, nvp63 lacks the amino acid motif (WxxΨF) otherwise present in the transactivation domain of vertebrate p53 family members. B Schematic representation of the nvp63, nvpVS53, nvpEC53, and human hup63 gene. All three p53-like genes were assembled according to the cDNA sequences determined by 5′ and 3′ RACE experiments. The nvp63 gene includes 14 exons (boxes) of which 11 exons encode the nvp63 protein (open boxes). 5′ and 3′ non-translated sequences are indicated by black boxes. The arrow depicts the transcriptional start site. The transactivation domain (TAD), the DNA binding domain (DBD), the oligomerization domain (OD), sterile alpha motif domain (SAM), and the transactivation inhibitory domain (TID) are boxed in yellow, red, green, blue and light grey respectively. Greek letters indicate alternative splice variants identified for hup63. ΔN indicates a cryptic transcriptional start site in intron 3 of the human p63 gene. Four introns interspersed within the DNA binding domain and one intron at the N-terminus of the tetramerization domain in nvp63 are at identical positions as in vertebrates. The nvpEC53 gene has acquired one more splice site within the 5′ end of the DNA binding domain and comprises in total more exons than the two other sea anemone genes nvp63 and nvpVS53. One of the introns conserved from nvp63 to human p63 within the DNA binding domain is absent in nvpVS53. The C-terminal variants of nvpVS53 identified so far are devoid of the canonical OD and putative TID. The observed alternative 3′ exon (exon 9) for nvpVS53 may translate a protein with altered DNA binding capacity. Alternatively, additional splicing patterns might exist during specific developmental stages.

The nvp63 cDNA (Fig. 5) encodes three major protein domains common to all vertebrate p53-like transcription factors: The nvp63 DNA binding domain (DBD) and the C-terminal oligomerization domain (OD) are highly conserved to the human p53 family member p63 (Fig. 6A). A lower but still significant similarity was observed between the N-terminus of nvp63 and the transactivation domain (TAD) at the N-terminus of vertebrate p63 and p53. In addition to these three typical transcription factor domains, two exons at the 3′ end of the nvp63 gene encode a putative transactivation inhibitory domain (TID) originally identified in hup63 [31]. The exons encoding the DBD and OD of nvp63 are separated by five introns at identical positions as in the hup63, hup53 and hup73 gene. Thus the core gene structure and parts of the protein domain structure of nvp63 have been well conserved from anthozoans to vertebrates (Fig. 6B). The gene structure of nvpVS53 or nvpEC53 is less similar to the vertebrate p53 gene family.

**Figure 7:**
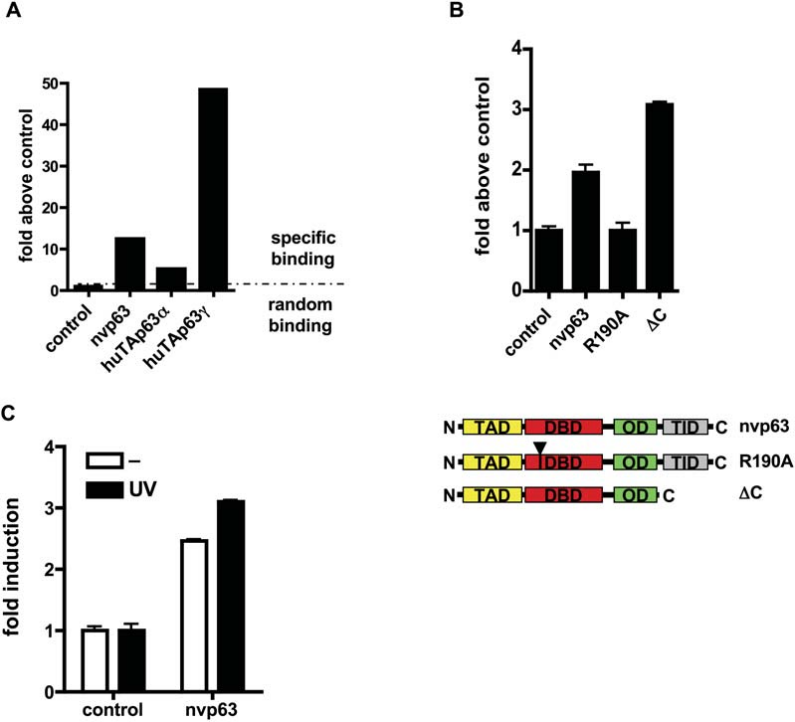
Nvp63 drives gene transcription in a heterologous expression system. A The direct binding for nvp63, human TAp63α, and human TAp63γ to the consensus p53 DNA binding sequence was determined *in vitro*. Lysates of SAOS-2 cells transfected with myc-tagged nvp63, TAp63α, or TAp63γ were incubated with biotinylated DNA encoding the canonical p53 binding sequence or random sequence. DNA-protein complexes bound to streptavidin were detected with an anti-myc-tag antibody. The value determined for the binding to the random DNA sequence was subtracted from the value obtained for the binding to the p53 binding sequence for each protein lysate. All obtained values are normalized to control. The dashed line indicates the signal obtained for the lysate with the random DNA sequence and the signal obtained with the p53 consensus DNA binding sequence. B Co-transfection of a luciferase reporter plasmid containing several repeats of the canonical p53 binding sequence allowed the determination of the level of nvp63-mediated transactivation. The mutation R190A in nvp63 abolished while the deletion of the C-terminal 34 amino acids (ΔC) induced reporter gene transcription. A schematic depicts the nvp63 protein and mutated variants. The transactivation domain (TAD), the DNA binding domain (DBD), the oligomerization domain (OD), and the transactivation inhibitory domain (TID) are boxed in yellow, red, green and light grey respectively. The black triangle and bar in the DNA binding domain indicates the position of the point mutation (R190A). C UV irradiation (1200 J/m^2^) of nvp63 expressing SAOS-2 cells increased the transactivation ability of nvp63. The fold induction in nvp63-transfected SAOS-2 cells is normalized to controls.

To test whether nvp63 binds to the consensus p53 RE, full length nvp63 protein with a C-terminal myc tag was over-expressed in the human cell line SAOS-2. Subsequently, the DNA binding of nvp63 protein was assessed in a DNA binding assay *in vitro* (Fig. 7A). Nvp63 bound to the canonical p53 RE with twelve fold higher specificity than to a random DNA sequence. The signal strength of the specific DNA binding was comparable to the signal strength determined for two hup63 protein variants, huTAp63α and huTAp63γ, which were identified previously [32], [33].

Transactivation reporter assays in a heterologous expression system with human SAOS-2 cells revealed functional conservation over more than 700 million years of evolution. Nvp63 induced the transcription of a reporter plasmid containing the canonical p53 binding DNA sequence when overexpressed in SAOS-2 cells (Fig. 7B). In hup53, DNA binding of the DBD depends on Arginine 176, a mutational hot spot frequently altered in cancer [34] that turns hup53 (R176) into a dominant negative mutant. Substitution of the identical Arginine (R204) in hup63 results in an autosomal dominant disorder characterized by ectrodactyly, ectodermal dysplasia, and orofacial clefts (EEC) [35], which is most likely due to a functional failure of hup63 to bind to its target DNA. Point mutants of the corresponding Arginine 190 in nvp63 (R190A) also failed to transactivate the luciferase reporter plasmid showing that proper DNA binding of the DBD in nvp63 is dependent on the same critical residues as in vertebrate p53-like proteins.

The DNA binding and transactivation capabilities of hup63 are induced when the outermost C-terminus is removed artificially [31], revealing it as a transactivation inhibitory domain (TID). Deletion of the last 34 amino acids in nvp63 (ΔC) increased transactivation activity in the reporter assay (Fig. 7B). The higher levels of transcriptional activation indicated the presence and functional preservation of a TID as a potentially regulatory element not only for human p63 but also for nvp63 mediated transactivation.

Next, we asked whether UV irradiation induces nvp63 transactivation activity in a heterologous expression system *in vitro*. Strong UV irradiation (1200 J/m^2^) of SAOS-2 cells transfected with the transactivation reporter system as described above increased nvp63 mediated reporter gene transactivation (Fig. 7C). This result suggests that the transactivation activity of nvp63 is responsive to UV irradiation. Because nvp63 itself is transcribed from a strong constitutively active expression plasmid, it is likely that post-translational modifications may regulate its activity by inactivation of the putative C-terminal TID.

In summary, nvp63 bound to the consensus DNA sequence recognized by hup53 and transactivated target gene transcription *in vitro*. UV irradiation increased nvp63-mediated target gene transcription *in vitro* and nvp63 localizes to early gametes that undergo UV-induced cell death *in vivo*. UV irradiation of adult polyps increased the signal of a protein complex bound to the p53 RE *in vivo*. These results suggested that UV-induced apoptosis of early gametes might be controlled by nvp63 *in vivo*.

**Figure 8:**
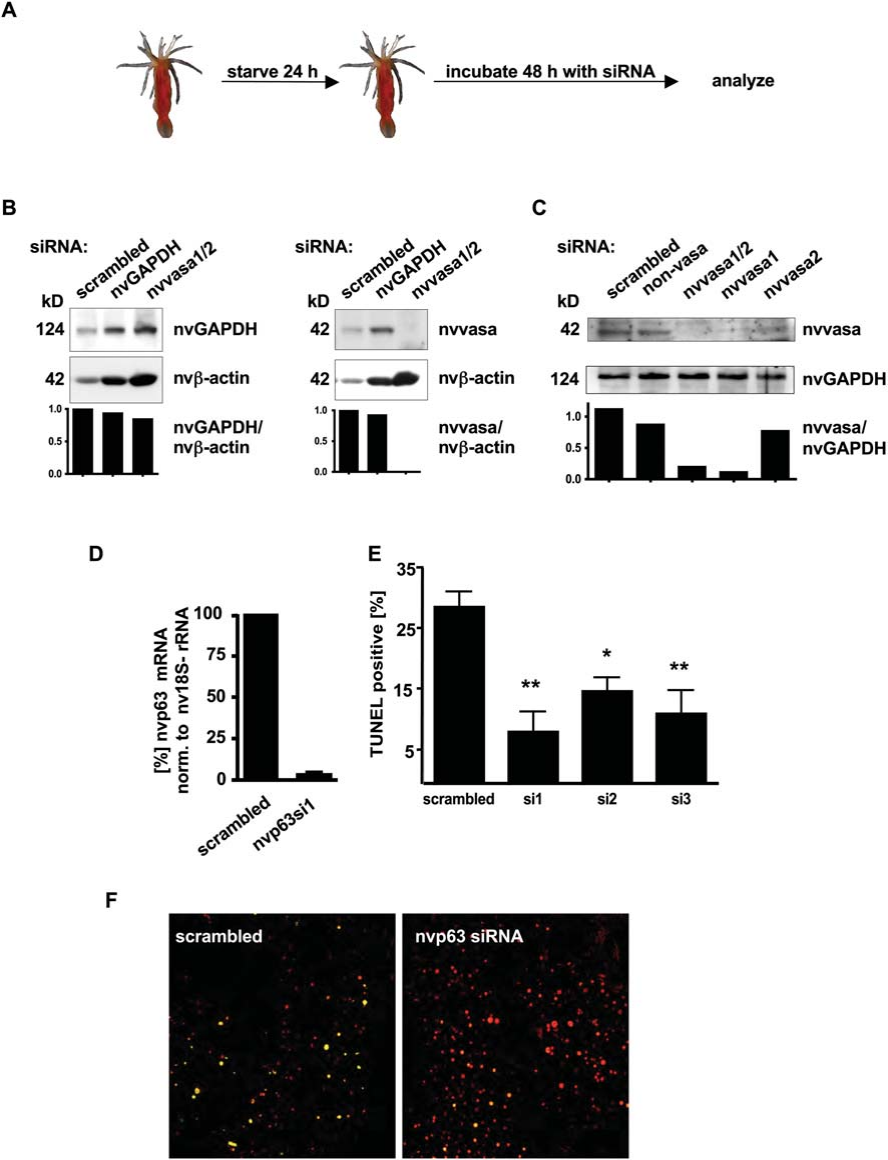
UV irradiation induced apoptosis is nvp63 dependent. A Schematic representation of the experimental protocol for the siRNA treatment of adult polyps. 24 h starved adult polyps were incubated in artificial sea water containing 10 µM double stranded siRNA for 48 h at room temperature. Subsequently, protein expression was analyzed by Western blotting. B Western blots depict the expression of nvvasa, nvGAPDH, and nvβ-actin after treatment of adult polyps with the indicated siRNA. Bar graphs indicate the amount of expression relative to cytoplasmic β-actin and after normalization to the scrambeled siRNA value. C Nvvasa1 is efficiently suppressed by RNA interference in the experimental setup. Adult polyps were incubated with a non-vasa siRNAs, a mixture of nvvasa1 and nvvasa2 siRNAs in equal amounts, or with nvvasa1 siRNAs or nvvasa2 siRNAs alone. The relative signal intensities normalized to nvGAPDH are visualized in the bar graphs below the Western blot signals. D Quantitative RT-PCR determined the knock down of nvp63 mRNA in adult polyps by the siRNA nvp63si1. The amount of nvp63 mRNA was normalized to nv18S-rRNA expression. E UV-induced apoptosis was determined at 12 J/m^2^ after incubation of adult polyps with the siRNAs indicated. Nvp63 specific siRNAs suppressed UV-irradiation induced death of gametes. * equals P<0.05, ** equals P<0.01. F Cell death was detected by TUNEL staining (green) on mesenterial tissue sections from adult polyps incubated with either scrambled siRNA or nvp63 specific siRNAs. Genomic DNA was visualized with Hoechst 3342 (colored in red).

To reveal the *in vivo* function of proteins in *N. vectensis* in general and of nvp63 in particular, we evaluated RNA interference as a strategy to knock down proteins in adult polyps *in vivo*. We tested the effectiveness of siRNAs targeting the ubiquitously expressed gene glyceraldehyde-3-phosphate-dehydrogenase (nvGAPDH) and the germ line-specific proteins nvvasa1 and its paralog nvvasa2. Adult polyps were incubated with double stranded siRNA molecules for 48 h and protein levels were subsequently determined by Western blotting (Fig. 8A). Incubation of adult polyps with nvGAPDH-specific siRNAs did not change nvGAPDH protein levels significantly when normalized to the control nvβ-actin signal (Fig. 8B, left). In contrast nvvasa-specific siRNAs efficiently eliminated all nvvasa protein (Fig. 8B, right). Nvvasa protein expression was assessed with an anti-vasa broad specific polyclonal antiserum first described by Chang *et al.* [36] which was successfully tested for detection of nvvasa in adult *N. vectensis* polyps by Extavour *et al.* [30]. Because two different nvvasa proteins (nvvasa1 and nvvasa2) exist and are potentially expressed in adult sea anemones we assessed the specificity of the RNA interference approach. The protein signal for nvvasa was nearly completely absent when nvvasa1 and nvvasa2 siRNAs together or nvvasa1 specific siRNAs alone were administered (Fig. 8C). In contrast, nvvasa remained detectable by the polyclonal antiserum following RNA interference with nvvasa2 specific siRNAs, suggesting that nvvasa1 is either the predominant isoform or preferentially detected by the antibody. As observed for other invertebrates like *C. elegans* [37], a nearly complete protein knock down can be achieved in the germ line, whereas somatic cells are relatively resistant to short term siRNA incubations.

In light of the findings that (1) the nvp63 mRNA is expressed in early gametes of adult polyps that undergo UV irradiation induced apoptosis and (2) the nvp63 protein bound to the conserved p53 DNA binding sequence and (3) nvp63 mediated gene transcription can be stimulated by UV irradiation *in vitro*, we tested whether nvp63 mediates UV irradiation induced gamete death in adult *N. vectensis* polyps, e.g. whether nvp63 knock down would reduce UV irradiation-induced apoptosis of early gametes. Adult polyps were incubated either with one of three different siRNAs against nvp63 or a control random sequence siRNA. Quantitative PCR determined a loss of over 90% of nvp63 mRNA molecules after 48 h of siRNA incubation (Fig. 8D). Subsequently, adult polyps incubated either with a scrambled siRNA or with nvp63 specific siRNAs were irradiated with 12 J/m^2^ UV light, a UV dose which is sufficient to induce apoptosis but low enough to reveal effects caused by the absence of nvp63 protein (Fig. 8E). Determining the number of apoptotic early gametes we found that only the nvp63 specific siRNAs but not scrambled siRNAs reduced the number of TUNEL positive cells in adult polyps (Fig. 8E, F). This experiment showed that knock down of nvp63 prevented the execution of the apoptotic program in early gametes. Therefore nvp63 is most likely required for UV-induced germ cell death in adult *N. vectensis* polyps.

Taken together these data provide intriguing evidence that in basal eumetazoans UV-induced cell death in the germ line is mediated by the human p63-like protein nvp63. Therefore activation of nvp63 after genotoxic stress in the germ line might serve as a mechanism to eliminate damaged gametes.

## Discussion

Here we report first clues for a molecular mechanism that controls apoptosis in a non-bilaterian animal, the sea anemone *Nematostella vectensis*. Cnidaria with a vast spectrum of species belong to the oldest animal phylae known and hold a key position at the base of the animal tree. Given the lack of p53-like proteins in unicellular eukaryotes, the discovery of p53-like molecules in cnidarians provides intriguing insight into the evolutionary origins of apoptosis controlling mechanisms.

A defining feature of all members of the p53 protein family characterized so far is that they bind to the canonical p53 responsive element. The core DNA binding domain and the canonical p53 RE can therefore be designated as an evolutionary conserved module. The discovery of a TID domain at the far C-terminus of nvp63 suggests the existence of a regulatory mechanism for nvp63 protein activity that has also been conserved through evolution. In contrast, nvpEC53 and nvpVS53 may have acquired functions other than that of nvp63 as concluded from the low protein sequence similarity between the three family members.

A molecular mechanism to protect the germ line is of major importance since deleterious mutations would boost the cost of reproduction if not selected against before fertilization. Therefore programmed cell death in *N. vectensis* gametes may act as a safeguard mechanism against too high mutational load in the germ line. Indeed germ cell death by defect is a recurring phenomenon observed in a variety of bilaterian species [2]. Consistent with the expression of nvp63 in the germ cell compartment of adult *N. vectensis* polyps, p53-like molecules have been found in the germ line of other species like *C. elegans* [38] and *M. musculus* [10] where they induce germ cell death upon γ-irradiation. The high functional conservation suggests that p63 may be a key protein within an evolutionary ancient germ line protective pathway.

A germ cell selective mechanism mediated by p53-like proteins on the one hand functions to maintain the integrity of the genome transmitted to the next generation. On the other hand, it may cause infertility in response to excessive or chronic genotoxic stress and may thereby threaten the survival of a species. The apoptotic response observed in the germ line of cnidarians is of special interest given that anthropogenic activities change the global climate with negative consequences for biodiversity [39]. One of the changes is elevated UV irradiation due to reduction or even depletion of the stratospheric ozone layer [40]. Elevated exposure to solar UV irradiation has a broad impact on marine species ranging from reduced photosynthetic activity of phytoplankton to defects in fish larval development [41]. Especially in combination with global warming this rise in UV irradiation has been proposed to alter the onset and magnitude of the reported biodiversity decline in coral reefs over the last decade [42], [43]. Despite extensive observations it is still under debate how cnidarians, to which reef forming corals belong, react to enhanced UV irradiation and cope with the increase of genotoxic stress. In particular, the molecular mechanisms involved in regulating the cellular stress response in the phylum Cnidaria have not yet been investigated.

**Figure 9:**
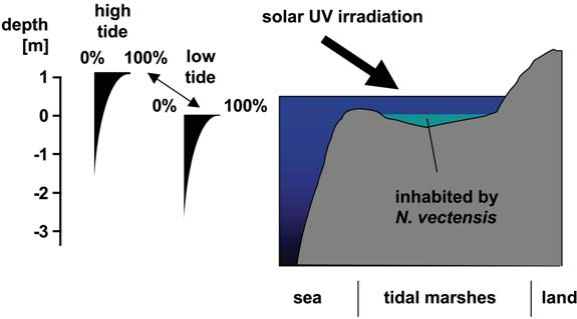
UV exposure in tidal marshes strongly depends on the sea level. The schematic qualitatively depicts the fluctuations in UV exposure for *N. vectensis* as a function of the sea level. Most of the DNA-damaging UV light drops within the first 1–2 meters to about 10% of UV irradiation present at the water surface. DNA damage caused by UV-irradiation decreases by several magnitudes within the first meters of coastal sea water.

Here we show that nvp63 may induce cell death in response to low doses of UV irradiation in gametes of *N. vectensis*. Natural UV irradiation of the normal habitat of *N. vectensis* may be an important genotoxic stressor: Although solar UV-irradiation is absorbed efficiently within the first meters of the surface water, in flat estuarine salt marshes–the natural habitat of *N. vectensis*–this protective barrier is subject to high variations due to water level changes for example along with the tide (Fig. 9). This leads to a more than 100-fold increased UV exposure of benthic organisms during low tide than during high tide. The amount of DNA damage induced by UV irradiation is inversely exponentially correlated to the height of the water column [44] and resident animals in shallow waters thus require highly inducible molecular response mechanisms to protect themselves against this variation in genotoxic stress.

Whereas the elimination of damaged gametes through nvp63-mediated apoptosis may contribute to maintain genome fidelity and stability and thus preserves a key feature of a stem cell, it may–as a negative consequence–lead to reduced fertility in response to high or chronic genotoxic stress. A decline in biodiversity subsequent to climate changes may thus not only be caused by death of developing or adult animals [45] but may also be a consequence of reduced fertility–a yet unrecognized correlation. Based on the results presented, further experiments in natural settings may take into account altered fertility of animals as a consequence of climate changes.

Nvp63′s protein structure, its localization in early gametes, and function suggest that the induction of apoptosis in the germ line upon genotoxic stress is a primordial function of the p53 family that existed already before bilaterians evolved. Further research will decipher the network activating nvp63 and identify components of this stress response pathway. The analysis of these signaling pathways and the regulatory molecules that decide over cell death after genotoxic stress in non-bilaterians will lead to a better understanding of the molecular evolution of DNA-protective mechanisms, which prevent malignant cell transformation and cancer in humans.

## Materials and Methods

### In vivo experimental procedures

Adult *Nematostella vectensis* polyps were collected in the Greater Sippewissett salt marshes at Cape Cod, MA, USA, or provided by the aquatic resources of the Marine Biological Laboratories in Woods Hole, MA, USA and cultivated in 1/3 artificial seawater (Tropic Marin), 2/3 freshwater under standard laboratory conditions [46]. All animals were fed every 24 h to 48 h with freshly hatched *Artemia salina* (Brineshrimpdirect, Utah). Experiments were carried out with adult *N. vectensis* at least 24 h after the last feeding.

### Cloning of the nvp63 cDNA, sequence analysis, and molecular modeling

A fragment of the nvp63 sequence homologous to *Mya arenaria* p63/p73 was identified in the NCBI EST database by BLAST sequence homology searches [47] and RT-PCR. The 3′ end and 5′ end were determined by rapid amplification of cDNA ends and all sequences were compared to WGS trace files and ESTs available at NCBI and www.stellabase.org [*48*]. The full length coding DNA sequence was amplified by RT-PCR from total RNA isolated from adult polyps. The nvp63 gene was assembled from WGS trace files by sequence comparison with the software package Lasergene (DNAstar, Madison). The nvp63 cDNA sequence (GI: DQ632751) and the annotation of the nvp63 gene (GI: BK005818) were deposited at GenBank. The mRNA sequences and the gene sequences for nvpEC53 and nvpVS53 were identified and assembled as described above (nvpVS53 cDNA GI: EF424410-EF424411; nvpEC53 cDNA GI:EF424412).

Sequence alignments were performed with ClustalW [49] and the following protein sequences were used for the phylogenetic tree construction with the PHYLIP package (Felsenstein, J. 2005. PHYLIP (Phylogeny Inference Package) version 3.6): *Homo sapiens* p53 (gi: 189478), *Homo sapiens* p63 (gi: 3695081), *Homo sapiens* p73 (gi: 2370175), *Mya arenaria* p63/p73 (gi: 7689272), *Drosophila melanogaster* p53 (gi: 8453175), *Caenohabditis elegans* CEP-1 (gi: 51038400). Phylogenetic trees were calculated with the NJ and UPGMA method and rooted subsequently. The protein sequences were aligned with CustalW and aligned sequences were bootstrapped 1000 times with SEQBOOT. Distance matrices of the protein sequences were calculated with PROTDIST and trees were assembled by NEIGHBOR. The bootstrap values are presented at the consensus tree (CONSENSE) determined.

### Transcriptional activation reporter assay

The nvp63 CDS obtained by RT-PCR was subcloned in pcDNA3.1A-myc/his vector and protein expression was detected in transiently transfected BHK cells with the monoclonal anti-c-myc antibody 9E10 by standard immunocytochemistry. SAOS-2 cells were transiently transfected with pRL (Stratagene), the p53 responsive element containing plasmid p53RE-pGL3, and the appropriate protein expression plasmid using Lipofectamine (Invitrogen). Transcriptional activation was determined in triplicates with the Dual-Glo Luciferase assay system (Promega). Luciferase expression was driven by the p53 DNA binding element of the human p21 gene and the resulting luminescence signal was normalized to the co-transfected Renilla plasmid.

### DNA binding and electromobility shift assay

To quantify DNA binding, SAOS-2 cells were transiently transfected with the pcDNA3.1A-myc/his plasmids encoding the cDNA of interest. Cells of one confluent well (12 well plate) were lysed in 30 µl binding buffer (20 mM Hepes pH 7.9, 20% Glycerol, 450 mM NaCl, 1% Nonidet P-40, 1 mM MgCl2, 0.5 mM EDTA, 0.1 mM 1,4-Dithiothreitol, Complete Protease Inhibitor Cocktail (Roche)) 24 h *post* transfection for 30 min at 4°C and remaining debris removed by centrifugation. 2 µl lysate was incubated in the presence of 0.5 µg poly dIdC with 2 pmol biotinylated double strand oligonucleotide coding either for the p53 DNA binding consensus (5′-/Bio/CCCGGGGCTGA ACATGTCTAA GCATGCTGAC CGGCCGG-3′) or random (5′-/Bio/CCGGCCGGAT GCCAAGCAGG TCCTTTAACA GCCCGGG-3′) sequence. Bound protein was detected with anti-myc-tag monoclonal antibody and quantified with the TransFactor Universal Chemiluminescence Detection Kit according to the manufacturer's recommendations (Clontech). The chemiluminescence signal was measured in a Wallac VICTOR2 1420 multilabel counter and each signal individually corrected for the background value obtained with the random DNA sequence.

For electrophoretic mobility shift assay, UV-irradiated (4800 J/m^2^, Stratalinker, Stratagene, 3 h recovery at room temperature) and control adult animals were completely dissolved in lysisbuffer (25 mM Hepes pH 7.6, 50 mM KCl, 1 mM DTT, 1 mg/ml BSA, 0.1% Triton X100, 20% Glycerol, 5 mg/ml EDTA-free complete Protease inhibitors (Roche, Bale, Switzerland), Phosphatase Inhibitor Cocktail Set I and II, 1∶100 diluted each (Sigma), 50 µg/ml poly-dIdC (Amersham), 50 µg/ml calf thymus DNA (Amersham)), sonicated, and lysates cleared from tissue and cell debris by centrifugation. Reverse complementary oligonucleotides harboring the canonical p53 (5′TAGACATGCCT AGACATGCCTA3′), the p63 (5′AGCTTGGACA TGCCCAGGCAG3′), or the random DNA sequence (5′ATGCCAAGCAG GTCCTTTAACA3′) were annealed and radioactively labeled by DNA polymerase I (NEB) mediated fill-in of 5′ guanosine overhangs (5 nt). 1 ng of annealed and radioactively labeled oligonucleotide was added to the protein lysate and incubated for 30 min on ice. Protein-DNA complexes were separated by native polyacrylamide gel-electrophoresis (4% Polyacrylamide, 0.3×Tris-Borate-EDTA buffer, 2% Glycerol, 0.05% Nonidet-P40) for 3 h at 300 V, 4°C. Gels were dried and exposed to a phosphoimaging plate.

### In situ hybridization

Adult polyps were relaxed with 3% urethane, fixed with 4% paraformaldehyde in 1/3 artificial sea water and embedded in tissue freezing medium (OTC, Jung, Nussloch, Germany). Longitudinal cryosections of 10 µm thickness were prepared and *in situ* hybridization was performed essentially as described [35]. Briefly, pretreated cryosections were incubated with sense and antisense digoxygenin labeled riboprobes over night at 60°C. Riboprobes covering the N-terminus of the nvp63 coding DNA sequence (nt 1-841) were generated by standard *in vitro* transcription in the presence of digoxygenin labeled-UTP. Hybridized cRNAs were detected with Alkaline Phosphatase conjugated antibodies to digoxygenin (Roche) and visualized by precipitation of NBT/BCIP (Promega). Sections were mounted in Mowiol (Sigma) and photographed with a Zeiss Axioscope2 microscope equipped with an Axiocam HRc.

### TUNEL assay and immunohistochemistry

Each adult polyp was placed in a droplet of medium in a Petri dish and UV-irradiated (Stratalinker, Startagene). Animals were kept for additional 8 h–12 h in the appropriate medium. Polyps were embedded in tissue freezing medium (OTC, Jung,) and transversally cut in 10 µm sections. Terminal deoxynucleotidyl transferase (TdT)-mediated dUTP nick end labeling (TUNEL) was performed with the *In Situ* Cell Death Detection Kit according to the manufacturer's recommendations (Roche). DNA was stained with Propidium Iodide and Hoechst 29934 (Molecular probes). At least 10 microphotographs for each condition were taken with a Leica SP2AOBS confocal microscope (Leica). Gametes were identified based on the high genomic DNA density and counted with Openlab software (Improvision). The percentage of apoptotic cells was calculated from the number of TUNEL positive gametes divided by all gametes identified on the section area. The standard deviation and the Student's t-test was calculated with Prism (Graphpad Software).

### RNA-interference mediated gene knock down

Adult polyps were incubated in 1/3 artificial sea water containing 10 µM siRNA for 24 h prior to UV irradiation. Double stranded siRNAs were either purchased from MWG Biotech (nvp63si-1: 5′–UGAAGUGACC UCAGUCUAA(dTdT)–3′) or generated with the Silencer siRNA Construction Kit (Ambion) (nvp63si-2: 5′–AAUGACGUCA AUGGAUUAUU A(UU)–3′; nvp63si-3: 5′–AAGAGUUCAC CAUUACGUUU C(UU)–3′; scrambled: 5′–AAUAGCAGAU UGCUAUGUAU A(UU)–3′). The GAPDH mRNA was blocked with the siRNAs nvGAPDHsi-1 5′-AACGAUCCCU UCAUCGACCU A(UU)-3′ and nvGAPDHsi-2 5′-AACUCUGGAG AAAGCCGGCU U(UU)-3′. Both cDNA sequences for nvvasa1 and nvvasa2 were assembled in full length from EST data available and their size calculated to 49.1 kDa for nvvasa1 and 51.7 kDa for nvvasa2. SiRNAs targeting these (nvvasa1-si: 5′-AAAGAGUCCA GACAAACGCU UUU-3′ ; nvvasa2-si: 5′-AAUGAAAAGA GAGACAGGUU AUU-3′) were designed according to the cDNA sequences inferred from assembled ESTs.

For Western blotting and protein detection polyps were lysed, tissue and cell debris were spun down, and proteins in the supernatant separated by SDS-acrylamide gel electrophoresis. Following Western blotting nvGAPDH was detected with a monoclonal antibody 4G5 (HyTest, Finland). This broadly GAPDH specific monoclonal antibody detects a single strong signal in whole cell lysates at a molecular weight at about 118 kD which is in good accordance with the predicted molecular weight of the nvGAPDH at 124 kD (Stellabase: SB_59557). Cytoplasmic nvβ-actin was visualized with the monoclonal antibody AC-15 (Sigma) which was raised against a synthetic cytoplasmic nvβ-actin N-terminal peptide with high homology to the N-terminus of the predicted nvβ-actin (Stellabase: SB_56628). The only signal detected in *N.vectensis* protein lysates runs at a molecular weight of 42 kD which is in good accordance with the molecular weight (41.8 kD) of the predicted nvβ-actin. The polyclonal rabbit antiserum directed against vasa protein was kindly provided by M. Akam. The specificity of the polyclonal antiserum for nvvasa protein was tested previously [30]. Bound antibodies were visualized with the appropriate HRP-conjugated secondary antibody and chemiluminescence reaction.

Knockdown of nvp63 was detected by quantitative PCR with the ABsolute™ QPCR SYBR-Green ROX mix (Abgene, UK) and a ABI PRISM 7700 sequence detector (Applied Biosystems, US) according to the manufacturer's recommendations. Results were quantified by comparative *C*_T_ method as suggested by the manufacturer.
